# The effect of training and awareness of subtle control on the frequency of hand hygiene among intensive care unit nurses

**DOI:** 10.1186/s13104-019-4635-z

**Published:** 2019-10-07

**Authors:** Zeinab Farmani, Marzieh Kargar, Zahra Khademian, Shahram Paydar, Najaf Zare

**Affiliations:** 1 School of Nursing and Midwifery, Shiraz University of Medical Sciences, Shiraz, Iran; 2 Community Based Psychiatric Care Research Center, School of Nursing and Midwifery, Shiraz University of Medical Sciences, Shiraz, Iran; 3 Trauma Research Center, Shahid Rajaee (Emtiaz) Trauma Hospital, Shiraz University of Medical Sciences, Shiraz, Iran; 4 Department of Biostatistics, Infertility Research Center, Shiraz University of Medical Sciences, Shiraz, Iran

**Keywords:** Infection control, Hand hygiene, Intensive care unit, Subtle control, Education

## Abstract

**Objective:**

This study aimed to determine the effect of awareness of subtle control after training on the hand hygiene compliance among nurses in intensive care units (ICUs). The study was conducted in two ICUs of a trauma center in Shiraz, Iran on 48 nurses. The nurses of one ICU were randomly allocated to the intervention and the nurses of the other ICU were allocated to the control group. All nurses were trained on hand hygiene. Then a fake closed camera television (CCTV) was visibly installed in the intervention group’s ICU, while the nurses were aware of it. The degree of compliance with hand hygiene was observed in both groups before and after the intervention. Data were gathered using a checklist based on the World Health Organization hand hygiene protocol and analyzed using SPSS 16 and the Chi square, Wilcoxon, Mann–Whitney U, and Independent T-tests, were performed.

**Results:**

The mean percentage of hand hygiene compliance in the intervention group after the intervention was significantly higher than before the intervention (p < 0.001). Additionally, the changes in the mean percentage of the intervention group was significantly higher than that for the control group (p = 0.001). The findings showed that a fake CCTV after training, installed in ICUs, can improve hand hygiene compliance.

## Introduction

Patients in intensive care units (ICU) are more susceptible to nosocomial infection so that the worldwide prevalence of infection among ICU patients was reported 29.5% [1]. The prevalence of nosocomial infections in Iranian public ICUs was estimated as 13.65% [2]. Health care worker’s hands are the most common means of transmission of health care-associated pathogens [3], that is why hand hygiene is recommended for all health care providers as the first step in the prevention and control of nosocomial infections [4]. Nurses have critical role in maintaining patient safety in ICUs [5]. Despite the importance of nurses’ role in reducing nosocomial infections and thus in reducing the hospitalization costs, complying with hand hygiene is low among nurses [6, 7] and health care workers in ICUs [7–9].

The World Health Organization (WHO) recommended multimodal hand hygiene improvement strategies including availability of alcohol-based hand rub and water supply, soap and towel, education, evaluation and feedback, reminders, and institutional safety climate [10]. Several studies have been conducted to evaluate different interventions, such as embedding alcohol-based solution at the bedside, e-learning [11, 12], structured training programs [13], providing feedback, and reinforcement [14], and patient’s participation [15]. Other studies investigated the multimodal interventions including strategies recommended by WHO and other ones [16].

Possessing sufficient knowledge is essential to the proper functioning of nosocomial infection control but it is not enough by itself. Thus, continuous training and motivation as well as monitoring and evaluation of nurses for nosocomial infection control are recommended [17]. Furthermore, timely feedback is one of the most important issues to encourage health care providers to comply with hand hygiene [18]. One of the ways to monitor hand hygiene practices is to use a video camera. In a previous study hand hygiene practices of health care providers after entering, exiting and remaining more than 60 s in patients’ rooms were evaluated by cameras and audited by 20 remote observers. In addition, feedbacks were given to health care providers [19]. However, their hand hygiene based on specific recommended moments was not reported. We hypothesized that a less costly intervention that includes individuals’ awareness of being monitored about hand hygiene could improve their performance. Therefore, this study aimed to determine the effect of awareness of subtle control after training, on the hand hygiene compliance among nurses in intensive care units (ICUs).

## Main text

### Methods

A semi-experimental study was conducted in two ICUs of a trauma center in Shiraz, Iran. The participants were 48 nurses in two ICUs who were working in the wards during the study and willing to participate. To determine the effect of subtle control by closed camera television on hand hygiene compliance, it had to be placed in one of the ICUs. Therefore, the nurses of one ICU were allocated to the intervention and the nurses of the other ICU were allocated to the control group by the lottery method. Data collection instruments included the demographic information form and the direct observation form. The checklist as a standard observation form proposed by WHO recommended for observing hand hygiene was utilized. The reliability and validity of the observation form, confirmed based on the Kappa coefficient of agreement on 10 samples, was reported to be 0.71 [6].

The study consisted of three phases. Before the intervention, the researchers directly observed the hand hygiene of the nurses in both the intervention and control groups when they were present at the patients’ bedsides for 15 min in each work shift (morning, afternoon, evening), while the nurses were not aware of this subtle control. Compliance to hand hygiene was observed and recorded on the five hand hygiene situations mentioned in WHO checklist. These moments included before touching a patient, before a clean/aseptic procedure, after body fluid exposure risk, after touching a patient, and after touching patient surroundings [20]. In next phase, the nurses in both the intervention and control groups were trained for 2 weeks. The educational interventions included face-to-face training, distribution of pamphlets and putting up posters in places that were visibly observable, such as on top of the sinks. The training issues mentioned in the pamphlets included: nosocomial infections and the important preventive role of hand hygiene, and methods and moments for hand hygiene. In the third phase, the fake CCTV was obviously installed in the ICU of the intervention group and the personnel were informed that their hand hygiene performance would be controlled via CCTV. The ethical committee recommended using fake CCTV. Hence, the real camera was not used. The researcher directly observed the hand hygiene compliance over a month as a pre-training performance using the checklist, considering the five hand hygiene moments. Similarly, the researcher directly observed the hand hygiene compliance over a month in the ICU of the control group, without the nurses being aware of this. In other words, the intervention group was trained, then the CCTV was installed and the nurses knew the CCTV had been installed for the subtle control. The control group was trained but did not know that they were being observed (Additional file 1). The hand hygiene facilities in both ICUs were the same so that in both ICUs an alcoholic solution was attached to the bottom of each patient bed; in addition, there were two sinks in the wards’ supply room and in the wards’ main areas. Hand hygiene by hand rub with alcoholic solution and hand washing was observed. Data from both ICUs were collected by checklist so that the performance of each group was compared before and after the intervention and compared with other group.

#### Data analysis

Data analysis was done using SPSS Software version 16. Descriptive statistics were used to describe the variables. The inter-group comparison of sex and marital status was performed by the Chi square test, and that for age and work experience was performed by independent T-test. In addition, to compare two groups in terms of the mean difference percentage of hand hygiene, Mann–Whitney U was used. The mean percentage of hand hygiene compliance in each group in terms of work shifts were analyzed by Wilcoxon test. A P-value less than 0.05 was considered statistically significant.

### Results

The intervention and control groups were homogenous in terms of age, gender, work experience and marital status. The mean age of nurses was 28.12 ± 3.18 years and most of the nurses (n = 36 0.75%) were female.

In both groups, the highest score of hand hygiene was after exposure to body fluids before and after the intervention (Table 1).

**Table 1:** The mean percentage of hand hygiene compliance before and after intervention in both groups

Hand hygiene compliance	Moments of hand hygiene compliance	Mean before the intervention (SD)	Mean after the intervention (SD)	P-value
Intervention group	Before touching a patient	24.2 (14)	32.5 (22.1)	< 0.001
	Before a clean/aseptic procedure	3.2 (7.4)	26.7 (19.5)	< 0.001
	After body fluid exposure risk	88.9 (21.4)	97.8 (7.2)	0.03
	After touching a patient	29.7 (24.3)	46.1 (20.6)	0.001
	After touching patient surroundings	11.6 (18.9)	17.1 (22.8)	0.07
	Total	24.5 (11.7)	41.9 (13.7)	< 0.001
Control group	Before touching a patient	45.6 (27)	54.8 (19.1)	0.14
	Before a clean/aseptic procedure	23.3 (22.3)	32.7 (19.1)	0.008
	After body fluid exposure risk	93.7 (15.2)	98.5 (19.1)	0.14
	After touching a patient	31.8 (24.8)	41.8 (17.1)	0.04
	After touching patient surroundings	14.6 (17.7)	20.6 (15.3)	0.04
	Total	34.1 (14.7)	41.8 (8.7)	0.004

The mean percentage of each group = $\frac{{{\text{The}}\;{\text{sum}}\;{\text{of}}\;{\text{hand}}\;{\text{hygiene}}\;{\text{percentage}}\;{\text{in}}\;{\text{the}}\;{\text{group}}}}{{{\text{Total}}\;{\text{number}}\;{\text{of}}\;{\text{participants}}\;{\text{of}}\;{\text{the}}\;{\text{group}}}}$

The Wilcoxon test was used

There was a significant difference between the two groups regarding compliance with hand hygiene before and after the intervention. The percentage of mean difference in the intervention group had a significant increase compared to the control group (p = 0.001) (Table 2).

**Table 2:** The mean difference percentage of hand hygiene between both groups

Moments of hand hygiene compliance	Control group	Intervention group	P-value
	Mean ± SD	Mean ± SD	
Before touching a patient	9.2 ± 28	8.2 ± 23.2	0.02
Before a clean/aseptic procedure	10.4 ± 18.5	23.5 ± 17.2	0.03
After body fluid exposure risk	5.1 ± 15.2	8.9 ± 20.6	0.36
After touching a patient	10 ± 22.5	16.5 ± 16	0.30
After touching patient surroundings	6.0 ± 14	5.5 ± 14.4	0.73
Total	7.7 ± 10.7	17.4 ± 6.9	0.001

The mean difference percentage = (mean percentage of hand hygiene compliance after the intervention) − (mean percentage of hand hygiene compliance before the intervention)

Mann–Whitney U was used

There was no significant difference among work shifts between the groups before the intervention. But after the intervention a difference was observed between the shifts in both groups. The mean percentage was higher in the morning shift, meaning that the hand hygiene compliance is higher in the morning (Table 3).

**Table 3:** The mean percentage of hand hygiene compliance between both groups before and after the intervention in terms of shifts

Shifts	Mean ± SD			P-value
	Morning	Evening	Night	
Intervention				
Before the intervention	28.5 ± 15.3	21 ± 11.8	2.7 ± 12.4	0.22
After the intervention	49.6 ± 15	38.4 ± 14.1	37.8 ± 20.9	0.001
Control				
Before the intervention	38.2 ± 16.9	30.7 ± 18.3	30.3 ± 16.2	0.08
After the intervention	47.2 ± 11.2	39.8 ± 9.5	37 ± 14.6	0.004

The Wilcoxon test was used

### Discussion

The results showed an increase in hand hygiene practice both in the intervention group who received training and were aware that they were under surveillance, and in the control group who only received training. Likewise, in Hang et al.’s study, the results indicated an increase in hand hygiene of healthcare workers after an educational program [21].

Despite the fact that subtle control was found as having a positive effect on hand hygiene after the intervention, it is not a completely satisfactory result. Contrary to this finding, in a previous study, video auditing and feedback increased hand hygiene from 10% pre-intervention to 81.6% post-intervention [19]. It is worth noting that, the duration of the mentioned study was longer than the current study and all the five hand hygiene situations recommended by WHO were not assessed. Based on current findings, looking for other ways to encourage health personnel to comply with hand hygiene, especially institutionalizing these values along with constant monitoring, is recommended in ICUs.

The hand hygiene compliance was low in both groups before the intervention. Similar findings are reported in the previous studies. The rate of hand hygiene from a total of 479 opportunities was estimated as 21.9% [6] and 12.8% of 500 opportunities in two Iranian studies [7]. In a study conducted in Riyadh, of the total of 3940 hand hygiene opportunities observed, 58% did not comply [8]. In another study in a teaching hospital in Kuwait healthcare workers hand hygiene compliance was 42.9% [22]. These findings indicate the importance of investigating interventions to improve hand hygiene.

In both groups, percentage of hand hygiene compliance after exposure to body fluids in all the shifts before and after the intervention was higher than all the other opportunities and this is consistent with the results of many other studies [10, 23]. Indicating that hand hygiene is perceived as more important when personnel consider it as a threat toward them, like when they are exposed to body fluids of patients. This observation can suggest the relative sensitivity of nurses toward prevention from catching the infection.

A low level of hand hygiene was observed after touching patient surroundings in both groups, similar to Randle et al.’s study [24]. Despite this fact, the role of surrounding environment in transferring pathogens has been verified, and cleaning and disinfection of the environment surrounding the patient is considered as one of the most important factors in reducing the acquired infections [25].

In this study, there was a difference among the shifts and the scores were higher in the morning shift in both groups. In Farbakhsh et al.’s study, likewise, hand hygiene was examined in two shifts and it was higher in the morning (26%) than in the evening shift (11%). Higher rates of hand hygiene in the morning shift may be related to the monitoring that takes place in the mornings by a head nurse and other authorities [6]. The importance of leadership in nursing profession has been discussed previously [26]. But contrastingly, in another study many personnel asserted that they are very busy especially in the morning when there is a greater workload that needs to be done in a short time; that is why they cannot take every opportunity to wash their hands or why they do not fully comply with hand hygiene [18].

### Conclusion

It can be concluded that the findings represent an increase in hand hygiene both in the intervention group who received training and were aware that they were being observed by the CCTV, and in the control group who only received training. However, the fact the mean difference percentage of compliance with hand hygiene between, before and after the intervention is higher in the intervention group suggests that, in general, awareness of subtle control results in more changes in compliance with hand hygiene. So, it is suggested that in ICUs the CCTV be used to observe hand hygiene compliance. Installing fake CCTVs can reduce some of the administrative problems associated with using real cameras. Given that the awareness of the subtle control of hand hygiene has a positive effect on nurses’ compliance. However, it is not still optimal. So alternative ways including institutionalizing these values along with permanent supervision, is recommended. Awareness of the subtle control has not had a significant impact on hand hygiene compliance after touching objects surrounding the patient. So there is a need to look for programs that improve the attitude of health personnel on hand hygiene compliance at this opportunity.

## Limitations

One of the limitations of the current study was the fact that the ICUs were assigned to intervention and control groups, and randomization based on nurses so that all of them have the equal chance to be in the intervention and control groups was impossible. Another limitation is about the possibility of the Hawthorne effect; the change in employees’ behavior as a result of observer’s presence. To reduce this effect in the ICU of the intervention group, the observer, who was one of the ICU nurses, directly observed the situations and completed the checklist in a subtle way. In the control group’s ICU, the checklist was also completed in a subtle way by the same nurse. The prolonged presence of the observer in the ICUs may have become habituated and reduced the likelihood of behavior change. It should be noted that WHO has introduced direct observation as the gold standard and the most reliable method in assessing the hand hygiene compliance [10].

## Supplementary information

**Additional file 1.** The steps of the study.

## Data Availability

Data are available upon request.

## Abbreviations

ICU: intensive care unit
CCTV: closed camera television
WHO: World Health Organization
