# Supplementary material for: The effect of training and awareness of subtle control on the frequency of hand hygiene among intensive care unit nurses
Source: BMC Res Notes. 2019 Oct 7;12:647. doi: 10.1186/s13104-019-4635-z (PMC6781344; doi:10.1186/s13104-019-4635-z)
Supplement: Supplementary file 1 — Additional file 1. The steps of the study. [file 13104_2019_4635_MOESM1_ESM.docx]

**Additional file 1:** The steps of the study.

All the nurses of two ICUs^*^ were selected (n=48)

Pre-test by direct observation (N=24).

Training on hand hygiene, during two weeks

Randomization by lottery method

Nurses of ICU1 were allocated to intervention group (n=24)

Nurses of ICU2 were allocated to control group (n=24)

Installing a fake closed camera television in the ICU1, visibly

Any other intervention

Post-test by direct observation (N=24).

Post-test by direct observation (N=24).

Data analysis (n=24)

Data analysis (n=24)

*Here they are called ICU1 and ICU2.
